# Draft Genome Sequence of *Marinobacter* sp. Strain LZ-6, Isolated from the Toxic Dinoflagellate *Alexandrium catenella*

**DOI:** 10.1128/MRA.00795-19

**Published:** 2019-09-12

**Authors:** Qiao Yang, Zhiwei Jiang, Xin Zhou, Xiaoling Zhang

**Affiliations:** a ABI Group, College of Marine Science and Technology, Zhejiang Ocean University, Zhoushan, Zhejiang, China; University of Southern California

## Abstract

Here, we report the draft genome sequence of Marinobacter sp. strain LZ-6, isolated from the cell culture of a toxic marine dinoflagellate, Alexandrium catenella LZT09. A total of 4,405 predicted protein-coding genes were revealed, including those associated with initial biosynthesis of the key intermediate of paralytic shellfish poisoning toxins (PSTs), namely saxitoxin, and with toxic compound extrusion.

## ANNOUNCEMENT

Marinobacter species are mesophilic, halotolerant or halophilic, and chemoheterotrophic bacteria most found in saline habitats, and many of them could be of potential interest for biotechnological and bioremediation applications due to their capacity to utilize hydrocarbon contaminants or to degrade plastic or radionuclides (1–4). However, few cultivable *Marinobacter* species are found to be associated with marine dinoflagellates, although members of *Marinobacter* are globally distributed in aquatic environments (5). Previously, *Marinobacter* sp. strain LZ-6 was isolated by serial dilution of the algal cells of toxic Alexandrium catenella and was plated on Difco marine 2216 agar (MA, USA) (6) and cultivated for 2 to 7 days at 28°C during the diversity investigation of the cultivable bacterial community associated with toxic dinoflagellates (7). A. catenella is a typical producer of paralytic shellfish poisoning toxins (PSTs) derived from globally widespread harmful algal blooms (8). For a better understanding of the bacterial roles in alga-bacterium interactions, including host PST biosynthesis, the genome sequence of strain LZ-6 was determined.

Strain LZ-6 was cultured in Difco marine broth 2216 medium at 28°C with shaking (180 rpm) for 2 days. Genomic DNA was extracted with a DNeasy UltraClean microbial DNA isolation kit, according to the instructions from Qiagen (MD, USA). The Illumina 2 × 250-bp paired-end library was prepared using a TruSeq DNA sample prep kit for Illumina (MA, USA), according to the manufacturer’s instructions, and then sequenced using a HiSeq 4000 platform (Illumina, CA, USA). Trimming and quality filtering with Trimmomatic v0.36 (9) yielded 5 × 10^6^ Phred Q30 reads. The read quality was assessed with FastQC v0.11.2 (http://www.bioinformatics.babraham.ac.uk/projects/fastqc). Genome assembly was performed with SPAdes v3.5.0 using the standard default settings (10). The assembled 55 contigs have 340× coverage with a length of 4,652,078 bp, a GC content of 57.0%, and an *N*_50_ value of 312,274 bp. Genome annotation through the NCBI Prokaryotic Genome Annotation Pipeline (PGAP) v1.2.1 (11) predicted 4,465 genes, which comprised 4,405 protein-coding genes, 53 tRNAs, 4 rRNAs, and 3 noncoding RNAs (ncRNAs).

Based on the genomic annotations, multiple homologous genes of four catalytic domains of the key initial *sxtA* gene involved in saxitoxin biosynthesis (12) were revealed, comprising those encoding methyltransferase (MTF; *sxtA1*), acetyltransferase (ATF; *sxtA2*), acyl-carrier-protein (ACP; *sxtA3*), and aminotransferase (ATF; *sxtA4*). Furthermore, genes encoding multidrug transporters of the ATP-binding cassette (ABC), multidrug and toxic compound extrusion (MATE) proteins (*sxtF* and *sxtM*), and major facilitator superfamily families were also identified (13).

### Data availability.

The GenBank accession number for the draft genome sequence of *Marinobacter* sp. strain LZ-6 is SWKL00000000. The BioProject and BioSample accession numbers for this project are PRJNA533500 and SAMN11445130, respectively. The raw sequence reads also have been deposited in the NCBI Sequence Read Archive (SRA) under the accession number SRR9678777.
